# Mathematics in modern immunology

**DOI:** 10.1098/rsfs.2015.0093

**Published:** 2016-04-06

**Authors:** Mario Castro, Grant Lythe, Carmen Molina-París, Ruy M. Ribeiro

**Affiliations:** 1 Universidad Pontificia Comillas, E28015 Madrid, Spain; 2 Department of Applied Mathematics, School of Mathematics, University of Leeds, Leeds LS2 9JT, UK; 3 Los Alamos National Laboratory, Theoretical Biology and Biophysics, Los Alamos, NM 87545, USA

**Keywords:** mathematical modelling, immunology, T cell, two-photon microscopy, T-cell receptor, diversity

## Abstract

Mathematical and statistical methods enable multidisciplinary approaches that catalyse discovery. Together with experimental methods, they identify key hypotheses, define measurable observables and reconcile disparate results. We collect a representative sample of studies in T-cell biology that illustrate the benefits of modelling–experimental collaborations and that have proven valuable or even groundbreaking. We conclude that it is possible to find excellent examples of synergy between mathematical modelling and experiment in immunology, which have brought significant insight that would not be available without these collaborations, but that much remains to be discovered.

## 1. Introduction

Mathematics has a long tradition in biology and medicine, going back at least to Gregor Mendel's work in genetics and Theodor Boveri's work on the nature of the chromosomes [1]. However, as the various subfields have become more specialized, and understanding of biological systems more detailed, modelling has often been dismissed or ‘regarded with suspicion, partly because much modelling seems an empty intellectual exercise that fails to deliver biological insight’ [2]. In immunology, the situation is not much different [3]. Some commonly cited reasons for the separation between experimental immunology and mathematical modelling are (i) the pace of discovery of new agents and new phenomena in the immune system, accompanied by new jargon, (ii) the rapid advance of technology, producing ever more data, and (iii) the contrast in academic environments, culture and terminology. In our view, these issues (at least the first two) are in fact reasons why mathematical modelling will become increasingly important in immunology (and other fields of biology) [4]. It is precisely because intuition is insufficient beyond a certain level of complexity that analysis of the immune system must become more quantitative [5–7].

As experimental data expose the complexity of the immune system, its nonlinearities [3], feedbacks [8], combinatorial complexity [9] and redundancies [10], it becomes ever more difficult to understand from a purely descriptive and qualitative viewpoint. On the other hand, big data and the so-called information explosion [11] have attracted attention as they promise automatic (computerized) solutions to biological and medical problems. The strategy behind this approach, referred to as *data-driven modelling* [12] or reverse modelling [13], is the use of statistical data analysis with the aim of creating novel knowledge. However, statistical analysis is only one of the many tools that mathematics can provide to immunology and, in general, cannot offer mechanistic explanations. Nor are computational models that include every known agent and interaction sufficiently well developed, yet, to replace animal testing. Current models, using differential equations, the theory of stochastic processes and agent-based simulations [14], are ‘often derived from phenomenology and guess work’ [13]. Their first benefit is that building the model forces the modeller to lay out all assumptions clearly. Thus, a mathematical model is an accurate description of current, incomplete, thinking, not a complete or accurate description of Nature itself.

Immunology is an excellent field for the application of mathematics, because it has a long tradition of important theories (clonal selection, immune networks, danger signals, … ) and great thinkers [15]. Still, there are impediments to a marriage of mathematics and immunology [3]. One is that the technical languages of the disciplines are very different. It would be useful for experimental immunologists to have some basic understanding of mathematical modelling, its usefulness and limitations; it is also crucial that modellers learn the required immunology and the experimental systems they are modelling. That is, we have to overcome the third problem identified above. On the other hand, there are examples of successful collaborations between experimental and mathematical immunologists. Often, but not exclusively, these partnerships are more fruitful when collaborations are initiated at an early stage of a given study.

Our aim here is not to review the impact of theoretical ideas in immunology, nor to provide a mathematical tutorial. We instead emphasize the role that mathematical (or computational) modelling has already played in immunology. We present examples from the literature of the contribution of mathematics to T-cell immunology under four headings: (i) generation of hypotheses, (ii) quantification of immunological processes, (iii) definition of observables to measure given an experimental objective, and (iv) reconciling disparate (or even conflicting) experimental results.

With our examples, we seek to illustrate the power that, together, mathematical and experimental immunology have to elucidate the behaviour of one of the most intricate systems that evolution has produced. To Dobzhansky's [16] statement that ‘nothing in biology makes sense except in the light of evolution’, we may add that nothing can be expressed quantitatively in science except in the light of mathematics.

## 2. Generating hypotheses

### 2.1. T-cell movement: directed or random?

T cells interact in lymph nodes (LNs) with antigen presenting cells (APCs) that display peptide–MHC complexes (small fragments of proteins bound to MHC molecules) on their surface. From the point of view of a naive T cell that spends a day or less in an LN, searching for an APC with a few cognate peptide–MHC complexes is a ‘needle in a haystack’ problem [17,18]. Based on twentieth-century understanding of cell types and the means by which cells communicate and home to different organs [17], derived from *in vitro* studies and snapshot images, the natural hypothesis was that, in the LNs, T cells are guided to their target APCs by chemokine-derived signals.

A new hypothesis emerged from three papers, published together in 2002, where Miller *et al.* [19], Stoll *et al.* [20] and Bousso *et al.* [21] reported *in vivo* imaging studies (using two-photon microscopy) in which individual T and B cells were resolved and tracked: the first direct *in situ* observations of their movement and interaction with dendritic cells (DCs). Movies were produced by stacking two-dimensional slices into three-dimensional images, then into series of three-dimensional images taken at regular time intervals, a minute or less apart. In the movies, ‘rather than directed motion in response to sustained chemokine gradients along structural pathways', T-cell motion is seen to be rapid, and described as ‘meandering’, ‘chaotic’, ‘random’ and ‘frantic’ [22,23]. The data available are the estimated positions of the centres of a set of labelled cells in three-dimensional space at a series of times: *x_i_*(*t*), *y_i_*(*t*), *z_i_*(*t*), where *i* labels the cell track and *t* labels time: *t* = 0, Δ*t*, 2Δ*t*, … , with Δ*t*, the time interval between images. It is well known from modelling of, for example, Brownian motion that, if the movement is indeed random, the means of *x_i_*(*t*)−*x_i_*(0), *y_i_*(*t*)−*y_i_*(0) and *z_i_*(*t*)−*z_i_*(0) are zero, but the mean of the displacement from the initial position, 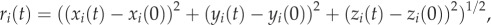
is not vanishing. When Miller *et al.* plotted the mean displacement against the square root of time, points fell ‘on straight lines, consistent with the hypothesis of random movement; the slope is the motility coefficient *M*’ [19,24]. Thus, in an opinion article in 2003, Cahalan *et al.* [25] argued for the new hypothesis ‘that lymphocytes migrate randomly within lymphoid organs, and that lymphocyte contact with APCs may be a stochastic process rather than one guided by chemokine gradient’. The ‘key point is that vigorous motility in a random walk characterises the behaviour of naive T cells *in vivo*’.

However, the situation is not so simple, because there can be subtle biases in cell tracks pieced together from two-photon imaging data [26], which are limited in both space and time. For example, directed movement in an environment full of physical obstacles (other cells and the endothelial reticular network) could still look approximately random. The best way to analyse these biases is to use mathematical models, which not only provide qualitative clues about the underlying mechanisms but, more importantly, in those cases where the true answer lies somewhere in the middle of two or more competing hypotheses, can describe quantitatively the way in which those different mechanisms are combined in the experiment, and identify additional experiments (for instance, inhibiting one mechanism) to test the validity of the model and gain a deeper biological insight.

Indeed, departures from a straight line in plots of mean displacement versus the square root of time are seen at sufficiently short and long times, indicating possible persistence in T-cell motion and an effect of confinement and finite imaging volumes. To analyse cell tracks in more detail and to compare them with theoretical predictions from random versus directed motion, the dependence on Δ*t* of the distribution of the apparent speed, 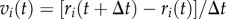, and the distribution of turning angles can be used [27–32]. These analyses have been applied to studies of the role of the LN reticular network [33–36], of the effect of localized chemotactic migration [37], and to extrapolate from the high density of labelled cells necessary for two-photon imaging to lower, more physiological, cell densities [38]. For example, careful mixed effects analysis is required to identify a small directed component of the motion of skin-resident T cells [39–41] towards a site of infection.

Not all T cells in LNs crawl vigorously; also noted in the early imaging studies were apparently stationary cells, clusters of cells and swarms of enlarged T cells that repeatedly come into contact with DCs bearing cognate antigen [24]. Bousso & Robey [42] estimated that seven new T cells per minute came into close contact with each individual DC, and extrapolated to conclude that each DC can contact about 500 different T cells in an hour. The description of Mempel *et al.* [43], in which T-cell priming by DCs in LNs occurs in three phases, has been very influential [22]. In the first of these phases, T cells contact antigen-bearing DCs intermittently and briefly. T-cell–DC contact durations then increase in the second phase and, in the third phase, T cells resume their motility, swarm in the local vicinity, begin the process of cell division and initiate interaction with B cells.

More sophisticated models of cell motion in the LN (such as those based on cellular automata, where cells move only according to stochastic local rules) can include these different phases, and try to use experimental data to calibrate the rates of T-cell interactions with other cells [36]. In yet more advanced computational models [44,45], called *cellular Potts* models [46,47], T cells are simulated as extended entities, that is, cells have shape (which can change over time) and sites that contact other cells, and occupy a physical space determined by their flexible cellular membrane. Each cell is assumed to minimize its surface energy. This gives rise to changes in the cell's configuration and to its movement over the course of time [48]. Extrapolating from experimental data, these models predict that an individual T cell can scan up to a hundred different DCs per hour [44].

These more complicated models allow detailed incorporation of random and directed components of the movement, with different hypotheses and taking into account the complexity of the LN environment. Moreover, they not only simulate the dynamics of cells in the LN, but one can also simulate the experimental measurements, including the effects of limited time and space resolution to clarify our interpretation of the experimental data. For example, it is possible to track single cells in a two-dimensional plane (or stack of two-dimensional planes) for a limited time, and plot their displacement. Thus, in this field of immunology, we find an example of a hierarchy of models, from simple functions to complex computer simulations, that provide more detailed analyses and insights into valuable experimental data and alternative hypotheses to explain T-cell migration in LNs. It is likely that both directed and random components are involved in T-cell movement, and this could depend on the type or state of the T cell. The final verdict is still unknown. Clearly, further rounds of observation–modelling–prediction–observation are needed to advance our understanding of how T cells move and interact with other cells, and how these interactions allow the specific pMHC–TCR interactions that initiate adaptive immune responses [49–51].

### 2.2. T-cell activation: how are signals integrated?

Adaptive immune responses to infection mediated by CD8^+^ T cells are characterized by the following time course: a subset of CD8^+^ T cells, those that are pathogen-specific, undergoes rapid proliferation, expanding the cell numbers of the given subsets, followed by population contraction owing to cell death and a return to homeostasis of the CD8^+^ T-cell population. T-cell activation and proliferation require a number of receptor-mediated signals. How are CD8^+^ T-cell stimulatory signals integrated and how do they relate to the strength of the T-cell response? A recent joint experimental and modelling effort by Marchingo *et al.* [52] has provided some answers, whereas previous efforts had been limited to qualitative approaches that do not predict the quantitative effect of altering a combination of stimulatory signals.

The current paradigm is that T cells respond to three signals: signal 1 (or *σ*_1_) is received by the T-cell receptor (TCR) upon ligand binding to peptide–MHC complexes, signal 2 (or *σ*_2_) is co-stimulatory and provided by APCs that express ligands, CD80 and CD86, that can bind co-stimulatory receptors (CD28 and CTLA-4) on the surface of T cells, and signal 3 (or *σ*_3_) is provided by cytokines (such as interleukin (IL)-2, IL-4 or IL-7). As mentioned above, there is an important difference between a qualitative hypothesis (*the three signals are needed*) and a quantitative/mathematical one (*which mathematical function describes the relative weight of these three signals*).

Previous work on B cells by the same authors led them to conclude that B-cell immune responses involve an automated programme of proliferation events, characterized by the number of total divisions and a return to cell quiescence [53–55]. These experimental studies also showed that the number of division events was influenced by the strength of B-cell stimulation. They hypothesized a similar programme for T cells, namely the final number of divisions during a CD8^+^ T-cell-mediated immune response is a (linear) function of the sum of signals, *σ*_1_, *σ*_2_ and *σ*_3_. The relevance of this hypothesis, if true, is the potential to predict therapeutic strategies of immunomodulation. Furthermore, the hypothesis naturally leads to the development of a quantitative (mathematical and computational) framework to relate the signals received by a given T cell to its behaviour during an immune response as a function of time.

Marchingo *et al.* [52] used the concept of *division destiny* (DD) of a T cell: the number of rounds of division before the cell returns to quiescence or dies. This concept was introduced in a paper describing the ‘cyton model’ of lymphocyte proliferation [53]. In this model, each cell has autonomous division and death processes in competition, corresponding (mathematically) to countdown clocks for these two fates (division or death). The start time for each clock is an independent random variable. The outcome for the cell is determined by whichever clock reaches zero earlier. DD is the maximum number of divisions allowed in the model [52,53].

A series of experiments allowed them to analyse how DD varies with different combinations of signals and to calibrate the cyton model to compute DDs [53,56,57]. Additionally, an *in vivo* experiment enabled them to test their hypothesis with a set of untrained data. Putting all these together, the authors then concluded that, both *in vitro* and *in vivo*, CD8^+^ T-cell-mediated immune responses imply two stages of regulation of T-cell DD. During the first stage, *σ*_1_, *σ*_2_ and *σ*_3_ additively programme a heritable number of division rounds before the first cell division. The second stage, which involves external stimuli, such as cytokines, can further increase the DD of a given cell.

The experimental approach of Marchingo *et al.* [52] makes use of transgenic mice with CD8^+^ T cells that only express one TCR, the OT-I receptor, crossed with fluorescent ubiquitination-based cell-cycle indicator red–green (FucciRG) reporter mice. The cell cycle is tightly regulated by specific proteins that are produced and degraded in a highly controlled fashion. The FucciRG reporter was developed by fusing red and green fluorescent proteins to two cell-cycle regulators, which are expressed at different times [58,59]. (More specifically, the reporter is based on the fact that the cell cycle is not only regulated at the transcriptional and post-translational levels, but also controlled by ubiquitin-mediated proteolysis. The authors harnessed the regulation of cell-cycle-dependent ubiquitination in order to develop two genetically encoded indicators for cell-cycle progression [58], hence the name, fluorescent ubiquitination-based cell-cycle indicator.) In this way, cells with this reporter (including the CD8^+^ T cells of the mice used) fluoresce red if they are in the *G*_0_/*G*_1_ phase, or green if they are in the *S*/*G*_2_/*M* phase of the cell cycle, and the cells can be classified as quiescent, recently divided or actively dividing. We should emphasize that the development of this reporter construct was a great technical achievement owing to the heroic efforts of Sakaue-Sawano *et al.* [58], opening doors for beautiful and important experiments [60]. Indeed, this is a very good example where new technology allows new insights into the biology, driving hypothesis formulation and model development.

Marchingo *et al.* [52] used the cyton model to fit data from FucciRG mice, allowing them to conclude that DD is around 10 generations (or divisions), yet different T-cell families differ in numbers, with differences up to three orders of magnitude.^1^

A second experimental model is an *in vitro* cell-trace-violet (CTV) division-tracking assay, to allow further exploration of the division progression. With this *in vitro* experimental system, the authors stimulated CTV-labelled OT-I-specific CD8^+^ T cells with a combination of signals *σ*_2_ and *σ*_3_ (see fig. 2 in [52]), and carried out a titration study for cytokine IL-2 (human). Again, fitting the data to the cyton model enables the authors to estimate the average DD of the population, to obtain a beautiful time course and dose–response curves based on the linearity hypothesis (Marchingo *et al.* [52] fig. 2*c,d*). Their *in vitro* CTV data support the hypothesis that summation of DD from multiple co-stimuli geometrically amplifies the T-cell response [52]. With these two sets of experiments, the authors make two key predictions. The first one is the additive nature of the T-cell stimuli *in vivo*, and the second one is that the second stage, which allows the extension of the DD of a given cell, will be more relevant away from the site of priming.

To test these predictions, the authors carried out a final set of experiments that involved co-transfer of T cells to mice infected with two different recombinant influenza viruses. One set of mice was infected with a virus expressing the low-affinity ovalbumin peptide *Q*_4_ (low affinity for the OT-I TCR expressed by the transferred cells), and a second set of mice was infected with an influenza virus expressing the high-affinity peptide *N*_4_ (high affinity for the OT-I TCR). The co-transfers involved two kinds of cells: wild-type (WT) OT-I CD8^+^ T cells and OT-I CD8^+^ T cells lacking the high-affinity IL-2 receptor (IL-2R*α* or CD25) (mutant), so that they cannot receive IL-2-derived cytokine signals. Thus, in total, the experiments generated four sets of data: (i) high TCR signal (*N*_4_ peptide) and high IL-2R signal (WT cells), (ii) high TCR signal (*N*_4_ peptide) and low IL-2R signal (mutant cells), (iii) low TCR signal (*Q*_4_ peptide) and high IL-2R signal (WT cells), and (iv) low TCR signal (*Q*_4_ peptide) and low IL-2R signal (mutant cells). The authors fitted the cyton model to data from (ii), (iii) and (iv) to predict the effect of each signal (in this case, *σ*_1_ and *σ*_3_) on the distribution of DD for the experimental conditions from (i). Based on that distribution, the authors were able to compute/predict the dynamics of the T-cell population over time, which showed good agreement with the experimental data.

This is one of the few (thus far) examples in which experiments and modelling have gone hand-in-hand and completed the full circle of: (i) experimentation, (ii) hypothesis generation, (iii) further experimentation, (iv) model development, (v) model prediction, and (vi) model validation with a final and independent set of experiments. The relevance of this study is multiple: first, it has managed to resolve long-standing discrepancies between *in vitro* and *in vivo* systems; second, it has provided a quantitative and predictive framework that underpins the time course of CD8^+^ T-cell responses for a combination of stimuli. Finally, this quantitative framework will allow further *in silico* testing for combinations of stimuli that might not be experimentally feasible now, yet relevant to the development of future immunotherapeutic strategies.

## 3. Defining what to measure

### 3.1. T-cell receptor excision circles to measure thymic output

To understand healthy homeostasis and disease dysregulation of the peripheral T-cell compartment, it is important to quantify thymic output [61]. However, there is no simple way to perform this quantification experimentally in humans (or mice). Ideally, recent thymic emigrants (RTEs) would express some surface protein with a short half-life that could be measured. This knowledge, together with the levels of cells expressing that protein in the periphery, would allow the calculation of thymic export, assuming RTEs did not proliferate, at least while expressing the identifier protein. This situation is the case in chicken, where the chT1 antigen on thymic T-cell precursors seems to fulfil this role [62]. In mammals, there is no such convenient marker, but it was proposed that one could use T-cell receptor excision circles (TRECs) to quantify thymic output [63,64].

TRECs are episomal DNA circles that are a by-product of TCR rearrangement. During T-cell development in the thymus, the *α* chain of the TCR is rearranged by recombination of its subcomponents (the variable and joining regions) on chromosome 14, which involves excision of the *δ* locus that resides between the *V* and *J* segment genes of the *α* chain on that chromosome. The excised DNA circularizes and contains specific sequences (so-called signal-joint and coding-joint) that are common on 70% of *αβ* T cells [63,64]. These TRECs have properties that make them good candidates for markers of RTEs with which one could quantify thymic output. In particular, they are stable [64,65], they do not divide [63], they are of exclusive thymic origin [63,64,66], they are identical in 70% of *αβ* T cells [67], and they mark RTEs in chicken [64].

Several groups used TRECs to quantify thymic output, especially in the context of HIV infection, because this infection leads to declining T-cell numbers and it was not clear what the contribution of a lower thymic output was [63,68,69]. In these studies, TRECs were reported as frequency of TREC^+^ cells among all CD4^+^ T cells (or, sometimes, naive CD4^+^ T cells or peripheral blood mononuclear cells (PBMCs)). It was found that TREC frequency decreases with age [63,68], that it is lower in HIV-infected individuals [63,68], although for many this frequency was in the normal range [68], and that it increases significantly in antiretroviral-treated HIV^+^ individuals, who have a lower than normal TREC frequency [63,68]. Another early study indicated that TREC frequency was a predictor, beyond viral load and CD4^+^ T-cell count, of the rate of HIV-1 disease progression [69]. These studies led to somewhat different conclusions, with some stating that RTEs and thymic output, as measured by TREC frequency, had an important role in HIV disease [63,69], and others concluding that ‘it is difficult to invoke thymic regenerative failure as a generalised mechanism for the depletion of CD4^+^ lymphocytes' and ‘we therefore caution against the use of such phenotypic markers as a direct indicator of thymic output’ [68]. This initial research was followed by a flurry of studies using TRECs to quantify thymic output [61,70–72].

One problem with the interpretation of TREC frequency is that it can vary owing to changes in both cells with TRECs (the numerator) and cells without TRECs (in the denominator). Thus, other dynamical processes besides thymic input into the periphery, such as T-cell proliferation or death, could influence the observed changes in TREC frequency. For example, T-cell proliferation of non-TREC-containing cells would lead to a decrease in TREC frequency, and it is well known that T-cell proliferation is increased in the context of HIV infection (see below). This problem was recognized and explicitly mentioned by the initial studies [63,64,68], and the assumptions about the importance of this issue affected the conclusions drawn by those authors.

This is a case where modelling can help clarify the interpretation of the data by identifying what to measure, and it can be argued that one must use a model to properly analyse the data. Exactly this was proposed in a seminal study, where a simple mathematical model was used to interpret TREC frequency data [73]. The model equations for TREC-containing cells (*C*) and total T cells (*T*) are [73]3.1
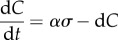
and3.2
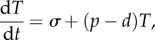
where *σ* is the output of the thymus, *α* is the fraction of cells exiting the thymus that contain a TREC and *p* and *d* are the rates of proliferation and death of T cells, respectively. The ordinary differential equations (3.1) and (3.2) are linear and, thus, most simple, yet allow one to explain the experimental data. In particular, with these assumptions for the dynamics of T cells, TREC frequency (*F* = *C*/*T*) is given by the division rule of differentiation and one obtains3.3
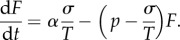
Equation (3.3) shows that the dynamics of TREC frequency depends explicitly on thymic output and also on the proliferation rate *p*, and cannot simply be interpreted directly as thymic output [74]. Hazenberg *et al.* [73] went on to analyse the predicted dynamics in the context of ageing, HIV infection and HIV treatment, and concluded that ‘loss of TREC in HIV-1 infection may primarily be caused by continuous hyper-activation of the immune system as reflected by increased cell division’ and ‘measurements of TREC content (…) fail to provide experimental evidence for thymic impairment’.

The contribution from the mathematical model was to clarify the assumptions made when thinking of TREC frequency as a direct measurement of thymic output, demonstrating that such interpretation of the data was abusive. However, the mathematical model also suggests that proper assessment of thymic output may be possible using TREC concentration (i.e. TREC^+^ cells per microlitre of blood), which is described by equation (3.1). This is clear from inspection of the equation, but was not realized until models of the system were developed. Measuring TREC per microlitre became an important variable in studies trying to understand the effects of thymic output [75–79], and also a relevant issue for model development [74,80–82]. In particular, equation (3.1) states that total abrogation of thymic output should result in an exponential decay in TREC concentration, and analyses of such data would allow estimation of the parameter *d* (the slope of that decay). Moreover, if we assume that TREC concentration is at an approximate steady state (*C*_0_) before abrogation of thymic output, then one can estimate thymic output as *ασ* = d*C*_0_. Thus, the model suggests an experiment of thymectomy (removal of the thymus). This experiment was performed on a macaque model of HIV infection, allowing direct quantification of the number of CD4^+^ and CD8^+^ T cells exported from the thymus (of rhesus macaques) per day [83].

The study of TRECs shows how development of novel experimental and technical capabilities can be leveraged even with simple mathematical models. It is clear that the model presented in (3.1)–(3.3) is a simplified description of complicated biological phenomena, and yet this approach was highly valuable to properly interpret new datasets.

## 4. Quantifying immunological processes

### 4.1. Estimating T-cell receptor diversity

The number of different TCRs that could be produced by the processes of DNA rearrangement that occur in the thymus is enormous, far greater than the total number of cells in a human body [84]. A first question is: how many different TCRs are found at any one time in one mouse or human? Let us denote this quantity by *N*(*t*). With the slight simplification that all the TCRs on the surface of any one T cell are identical, the T-cell repertoire can be divided into clonotypes, each clonotype characterized by a unique TCR. If *n_i_*(*t*), *i* = 1, … ,*N*(*t*) is the number of T cells expressing TCR of type *i* at time *t*, then we can pose a second question: what is the distribution of values of *n_i_*(*t*)?

The TCR is formed in the thymus by pairing a larger *β* chain and a smaller *α* chain. The DNA subset that encodes the *β* chain, TRB, is formed by rearranging V, D and J gene segments, plus additional nucleotide rearrangements at V–D and D–J junctions. TCRA, which encodes the *α* chain, rearranges with V and J only. Assuming that the TCR nucleotide sequence provides a unique molecular tag for each clonotype, the total number of distinct TCR sequences in a body is equal to *N*(*t*). A new field of immunosequencing has emerged with technologies designed to sequence TCRs [85]. Millions of TCR sequences can be amplified in a single multiplex polymerase chain reaction (PCR), prepared and then read in parallel from a single sample. The distribution of VJ gene segment usage can be measured with flow cytometry [86,87], and used to track changes with age and between individuals [88,89].

In 1999, Arstila *et al.* [90] published a first estimate of the number of distinct TCR*β* chains in a sample of 10^8^ T cells from a healthy donor. By sequencing only DNA from cells that use VB18 (estimated to be 0.8% of T cells), VJ1.4 (estimated to be 3% of T cells) and with a CDR3 length of 12 amino acids (estimated to be 9.3% of T cells), they were able to identify 17 different TCR*β* chains. Extrapolating, they concluded that the blood sample contained 17/(0.008 × 0.03 × 0.093) = 0.8 × 10^6^ distinct TCR*β* chains. Fifteen sequences from the same sample, restricted to VB16, VJ2.2 and CDR3 length of 13 amino acids, gave a similar estimate of TCR*β* diversity, close to 10^6^. Subsequent studies have tended to restrict themselves to analysing the *β* chain, believed to contain the majority of TCR diversity. The estimate of 10^6^ TCR*β* chains in human blood, first obtained by audacious extrapolation, has been backed up by more recent studies [91].

In 2000, Casrouge *et al.* [92] analysed mouse spleens [93]. The same mathematical extrapolation as that just described, from the subset of VB10, VJ1.2 cells with 10-amino-acid-long CDR3, yielded an estimate of half a million distinct TCR*β* chains. They also estimated that the combination with the TCR*α* chain contributes a factor of more than two to the diversity, thus concluding that a mouse spleen contains about 10^6^ clones of about 10 cells each. In mice, different T-cell subsets can be compared, and the effects of infections and immunization on the repertoire can be tracked [94–97]. Current single-cell approaches [98] are able to provide information about the promiscuity of the *αβ* pairing, which is needed to establish the true extent of TCR clonal diversity.

Freeman *et al.* [99] identified 33 664 distinct TCR*β* sequences in a pool of cells from 550 people. Recent studies, which were able to directly count large numbers of sequences, have identified 10^6^ distinct TCR*β* chains in a sample of blood from one person. It is possible to use ecological ‘missing species' analyses [100–102] to estimate the total number of distinct TCR*β* chains in the blood. Estimates range from a few million to as high as 100 million [100,101,103]. Li *et al.* [104] isolated PBMCs and extracted genomic DNA from 10 ml of blood from two rhesus monkeys. They identified nearly a quarter of a million unique CDR3 amino acid sequences in the sample.

**Figure 1.**
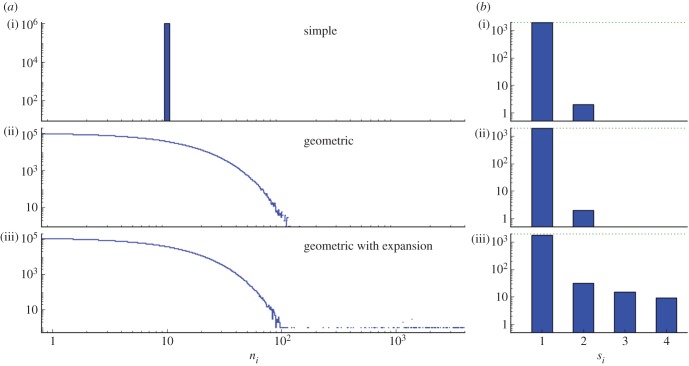
Simulated sampling from repertoires. In each of the three simulated examples shown, a sample of 2000 cells is selected from a repertoire that contains 10 million cells divided into 1 million different clonotypes. (*a*) The three examples have different distributions of clonal sizes, *n_i_*. In the first, every clonotype consists of 10 cells (*a*(i)). In the second, the number of cells per clone is drawn from a geometric distribution with mean 10 cells (*a*(ii)). The third example is similar to the second, but 200 ‘expanded’ clonotypes have sizes drawn from a geometric distribution with mean 1000 cells (*a*(iii)). (*b*) Histograms of the samples (of 2000 cells) from each repertoire. The number of cells, *s_i_*, of a clonotype, *i*, that is present in the sample is most often equal to 1. Only in (*b*(iii)), where there are expanded clonotypes in the repertoire, are there more than two cells of some clonotypes in the sample. (Online version in colour.)

Counting experimentally the total number of distinct TCRs in a body may never be possible, but even if it becomes possible, it will not fully characterize the T-cell repertoire, i.e. the number of cells of each clone. In fact, (deep) sequencing does not provide a direct measurement of the distribution of values of *n_i_*, because the number of times a sequence is found depends on mRNA copy numbers inside cells and on biases in PCR amplification [105]. Sequencing one cell at a time is an attractive alternative, but is currently limited in practice to a few hundred cells per experiment. Computational models, on the other hand, are rapid and cheap and may provide the only alternative to properly quantify the distribution of clonotype sizes. Here, at least two approaches are possible: (i) using statistical models to compare distributions and define observables and (ii) developing mechanistic models of cell division and death to establish what parameters influence the steady-state distribution of clonotype sizes. For example, in the first approach, we could define three ways of allocating 10^7^ cells (of a mouse) into the estimated 10^6^ different clonotypes [92], as illustrated in figure 1. We could then take samples of 2000 cells from each distribution, to replicate an experimental protocol, and compare the resulting histograms from experimental data and the computational model. In the case shown, most of the TCRs that are present in a sample are found only once. The presence of two or more cells of the same TCR clonotype in such a sample is evidence that there are clonotypes with large numbers of cells in the repertoire (e.g. expanded in response to antigen).

Whatever statistically stationary set of *n_i_*(*t*) is compatible with human (or mouse) experiments results from a balance between T-cell generation (in the thymus), division and death. Thus, mathematical models of the dynamics of the T-cell population, in which the *n_i_*(*t*) are functions of time, governed by thymic production of new T-cell clonotypes, and division and death of existing cells in the periphery have been proposed [106–110]. Often these models simulate ecological competition within and among clones for TCR signals and other resources (e.g. IL7) to study what factors are more important in determining the distribution of T-cell clones. Moreover, using estimates for the basic processes (of thymic generation, division and death) from human and mice, the models allow comparison of predictions of the distribution of clonotypes in those two cases. One such model predicts a much larger number of clonotypes than estimated experimentally so far [110]. The model could be used to produce figures similar to figure 1 to start comparing the predictions with the data, and if needed the model could be refined to better describe experimental data. In summary, as well as requiring statistical models to deduce the size of the T-cell repertoire from experimental measurements, mathematical modelling is needed to understand how homeostasis of that repertoire is attained and maintained.

### 4.2. Quantifying thymic development of T cells

A beautiful example of how experimental data together with a mathematical model allows us to quantify immunological processes is provided in [111]. This paper provides an excellent lesson on the role that modelling can play in quantifying immunological process; in doing so, new light is shed into the mechanisms that are at the heart of the given immunological process.

Cells of the mature *αβ* T-cell repertoire arise from the development in the thymus of bone marrow precursors (or thymocytes). *αβ* T-cell maturation is characterized by the expression of thousands of copies of identical *αβ* TCRs and the CD4 and/or CD8 co-receptors on the surface of thymocytes. The overall maturation stages of a thymocyte are (i) double negative (DN) (TCR^−^, CD4^−^ and CD8^−^), (ii) double positive (DP) (TCR^+^, CD4^+^ and CD8^+^), and (iii) single positive (SP) (TCR^+^, CD4^+^ or CD8^+^). Thymic APCs provide the appropriate microarchitecture for the maturation of thymocytes, which ‘sense’ the signalling environment via their randomly generated TCRs. Naive CD4^+^ and CD8^+^ T cells that populate the immune system derive, thus, from the SP CD4^+^ and CD8^+^ thymocytes, respectively, that were successfully selected and exited the thymus into the periphery. The observed ratio of naive CD4^+^ to CD8^+^ T cells is about 2 : 1; that is, there are more CD4^+^ helper T cells than CD8^+^ cytotoxic T cells in the peripheral pool [81,112]. A long-standing immunological question is the origin of this asymmetry in CD4^+^ and CD8^+^ T-cell numbers and, in this paper, the authors set out to study thymic development in search for clues to this bias [111].

**Figure 2.**
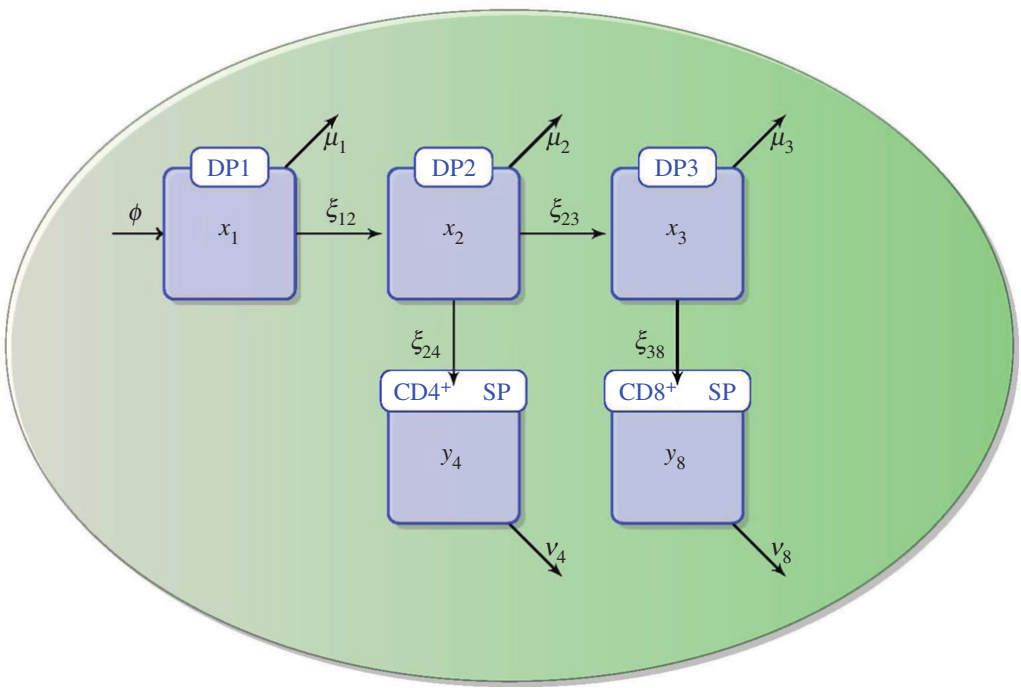
Mathematical model of thymocyte development as introduced and developed in Sinclair *et al.* [111]. Thymocytes enter the DP1 stage from DN cells with influx *ϕ*. At the DP1 stage, thymocytes can either die with rate *μ*_1_ or differentiate to the DP2 stage with rate *ξ*_12_. At the DP2 stage, thymocytes can either die with rate *μ*_2_, or differentiate to the DP3 stage with rate *ξ*_23_, or to the SP CD4^+^ stage with rate *ξ*_24_. DP3 thymocytes can either die with rate *μ*_3_ or differentiate to the SP CD8^+^ stage with rate *ξ*_38_. CD4^+^ SP thymocytes die or exit to the periphery with a total loss rate *ν*_4_, and CD8^+^ SP do so with rate *ν*_8_. (Online version in colour.)

Their approach combines a carefully designed experimental study and a mathematical model that allows them to quantify differentiation and death rates of different subsets of thymocytes during their development (figure 2). Their results are not only the estimation of these parameters, but also the ability to discriminate between two hypotheses for the observed asymmetry in CD4^+^ and CD8^+^ T-cell numbers. The authors set out to answer the question: what is the source of the asymmetry between the CD4^+^ and CD8^+^ T-cell lineages during thymic development? The experimental study is based on their ability to synchronize cohorts of thymocytes *in vivo* to follow their kinetics as they develop and are being selected. The mathematical model is based on a set of deterministic ordinary differential equations that describe the dynamics of the thymocyte populations from the DP stage (figure 2). The model includes five populations (DP1, DP2, DP3, CD4^+^ SP and CD8^+^ SP), where the DP population is subdivided into three distinct temporal stages of development (DP1, DP2 and DP3), which had previously been experimentally identified based on the surface expression of TCRs and CD5 (see fig. 1 in [111,113]). The model also includes the rates of maturation from one stage to the next (*ξ*_12_, *ξ*_23_, *ξ*_24_ and *ξ*_38_), and loss (*ν*_4_ and *ν*_8_) or death (*μ*_1_, *μ*_2_ and *μ*_3_). Loss is used to jointly describe death or migration in the SP thymocyte compartments. A migration term is included, *ϕ*, to represent the influx of cells into the DP1 stage from DN thymocytes. Finally, the mathematical model does not include cell proliferation as recent estimates suggest that there is no proliferation during the DP stage, and at most one round of proliferation during the SP stage [111,114].

Quantifying and deciphering the mechanisms/sources of asymmetry is a big challenge! At least two different hypotheses can explain the thymic origin of the bias:

— at the DP1 stage, there is an asymmetry in the number of DP thymocytes with a TCR that can bind to MHC class I versus MHC class II, or
— during the DP or SP stage, there exists some asymmetry in either maturation or loss processes (migration to the periphery or death).

Three sets of experiments are carried out to identify the source of the CD4/CD8 bias [111]. The first (or control) experimental assay arrests thymic maturation at the DP stage to then switch it on, and thus T-cell development can be followed during 10 days in a synchronized way. Cell numbers were analysed with flow cytometry at days 0, 1, 2, 3, 4, 5, 6, 7, 8, 9 and 10. The second experimental assay is exactly as the first, but with MHC class-I-deficient mice, so that only CD4^+^ T cells can be selected. Finally, the third experimental assay is performed, with MHC class-II-deficient mice, so that only CD8^+^ T cells can be selected. In this way, the authors can simultaneously and independently study the development of CD4^+^ and CD8^+^ T cells. That is, the experiments with deficient mice allow them to understand lineage-specific maturation and death rates from the DP2 stage under conditions of isolated development.

Given the experimental data and the mathematical model, the authors obtain estimates for the parameters of the model. The most striking differences appear in the DP2 compartment. For example, for the control experiments, they estimate *ξ*_24_ = 0.14 per day and *ξ*_23_ = 0.07 per day, whereas for the MHC class-I-deficient case 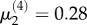 per day and 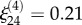 per day, and for the MHC class-II-deficient case 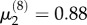 per day and 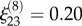.^2^ This indicates that a source of asymmetry is the difference in death for the CD4^+^ and CD8^+^ thymocytes at the DP2 stage. It is at the DP stage when the commitment to either CD4^+^ or CD8^+^ lineage is decided. Furthermore, they introduce the dimensionless parameter *s*, or selection efficiency, for any of the DP compartments as the ratio between the maturation rate and the sum of the maturation and death rates. For example, for DP2 thymocytes in the case of MHC class-I-deficient T-cell development, the selection efficiency is given by4.1
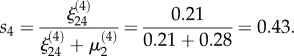


In the case of MHC class-II-deficient T-cell development, the selection efficiency is given by4.2
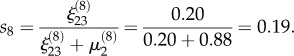
Thus, not only is the CD8^+^ lineage at a disadvantage in terms of death rates during the DP2 stage, but also the selection efficiency is lower than that of the CD4^+^ lineage. On the other hand, at the DP1 stage, no significant differences are observed between the death *μ*_1_ and maturation rates *ξ*_12_ estimated for the three different experimental conditions: control, MHC class-I- and MHC class-II-deficient mice.

Taken together, experimental observations and parameter estimates for MHC-deficient mice, the authors can identify that the source of CD4 enrichment takes place at the DP2 stage, given the differences in both death rates and selection efficiencies. Thus, one can now discriminate between the two hypotheses, as the data and the quantification do not support a greater abundance of MHC class-II-restricted thymocytes than MHC class I at the DP1 stage (first hypothesis).

## 5. Reconciling experimental results

### 5.1. Quantifying T-cell proliferation

HIV-1 infection results in depletion of CD4^+^ T cells, but it is not clear what dynamical processes are responsible for this cell loss. One hypothesis is that reduced production from the thymus (see §3.1) causes a decline in cell numbers. Other possibilities are changes in proliferation and death rates of CD4^+^ T cells. Several clever experimental assays have been developed to analyse proliferation rates of T cells in humans, allowing comparison of healthy and HIV^+^ individuals.

One of the first measurements of CD4^+^ and CD8^+^ T-cell proliferation was based on the length of their respective telomeres. These are segments of repeated nucleotide sequences at the end of chromosomes, which protect them during replication, because copying of the DNA cannot proceed to the very end of the molecule, since the copying enzymes fall off. Thus, at every cell division, chromosomes lose some nucleotides at the end and, without telomeres, information would be lost (i.e. genes could be truncated). Proliferation of T cells (and others) leads to shortening of the telomeres. Wolthers *et al.* [115] used this idea and measured telomere length over time (up to 10 years) in healthy and HIV-infected individuals, in both their CD4^+^ and CD8^+^ T cells. They found stable telomeres in both subsets of healthy people, whereas in HIV-infected people there was no change in the length of telomeres in CD4^+^ T cells, but a large decrease in this length in CD8^+^ T cells. This was interpreted as providing ‘evidence that turnover in the course of HIV-1 infection can be increased considerably in CD8^+^ T cells, but not in CD4^+^ T cells' [115].

A second method to assess T-cell proliferation is based on the expression of the Ki-67 antigen, which is a protein expressed only during cell division. Thus measuring the fraction of T cells bearing this antigen gives a snapshot of the proliferative state of these cells. Analyses of this marker showed that both populations of CD4^+^ and CD8^+^ T cells have increased expression of Ki-67 in HIV-1 infection compared with healthy age-matched controls [116]. Although there was large person-to-person variability, there was a significant correlation between the fractions of Ki-67^+^ CD4^+^ T cells and Ki-67^+^ CD8^+^ T cells, as well as between Ki-67^+^ CD4^+^ T cells and HIV-1 viral load in infected people, indicating that both CD4^+^ and CD8^+^ T-cell subsets have increased turnover in the setting of HIV-1 infection [116]. These results were confirmed and extended in another study that analysed Ki-67 expression before and after antiretroviral treatment for HIV-1 infection [117], which showed that not only was proliferation increased in relation to healthy controls, but this proliferation also declined rapidly with the initiation of treatment [117].

In part, because the methods described are indirect and, in part, because there were relevant discrepancies among the results, a direct measurement of T-cell turnover was applied in the context of HIV-1 infection [118]. This method is based on infusion of deuterated glucose (i.e. glucose where hydrogen has been partly replaced by deuterium) to volunteers. This deuterated glucose will be incorporated in newly synthesized DNA of proliferating cells, in effect labelling them. Sampling T cells from blood after infusion and measuring the fraction of cells labelled with deuterated glucose allows the quantification of T-cell turnover. However, even this ‘direct measurement’ needs some mathematical model to correctly interpret the labelling data. In the first study of HIV-1 infection using this technique, with a 2-day infusion of deuterated glucose, the increase in proliferation of both CD4^+^ and CD8^+^ T cells during HIV-1 infection was confirmed, but, surprisingly, it was found that both proliferation rates were even more increased during successful therapy (i.e. suppressing viral load below detection) [118].

**Figure 3.**
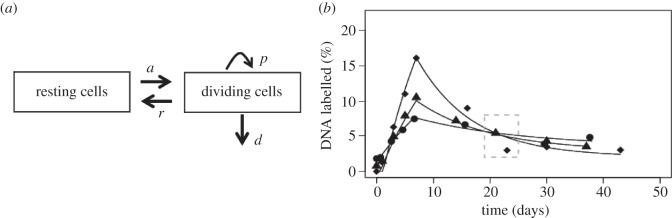
Model used to analyse deuterated glucose labelling of dividing T cells. (*a*) Schematic of the model showing the populations of resting and dividing cells, their interconversion (activation, *a*, and reversion, *r*), proliferation (*p*) and death (*d*) of dividing cells. (*b*) Example of model fit to data for the labelling of cells in one HIV-1-infected person before therapy (diamonds), after short-term therapy (triangles) and long-term therapy (circles). Therapy leads to lower label incorporation, indicating slower turnover; however, if one measures incorporation only after 20–25 days (dashed grey box), there are higher levels of labelling after long-term treatment, which would suggest elevated turnover. The model clearly shows that it is the slope of incorporation or loss of label that contains the relevant information about turnover, not the labelling level at any one time.

Altogether, these experimental studies resulted in contradictory conclusions, which is a good indication that a modelling approach can be very useful. Initial modelling results suggested that a longer infusion of deuterated glucose, leading to higher T-cell-labelled fractions, together with time courses of labelling, would be more informative. A study was devised with 7-day continuous infusion of deuterated glucose, followed by sampling up to six weeks post-infusion [119]. These data were analysed with a new model allowing for cell activation, proliferation and death (represented in figure 3) [120]. The equations for the resting cells, *R*, and activated cells, *A*, are5.1
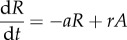
and5.2
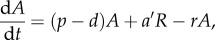
where *a* is the activation rate from a compartment of truly resting cells (with an allowance for clonal activation, hence the *a*′), *r* is the rate of return to rest from the activated (dividing) state, and *p* and *d* are the proliferation and death rates of activated (dividing) cells, respectively (a full analysis of the model is provided in [121]). With the help of this model, it was established that proliferation of both CD4^+^ and CD8^+^ T cells was increased during HIV-1 infection, and it quickly reduced after the initiation of antiretroviral treatment [119,120]. The estimates of proliferation from the model were highly correlated with Ki-67^+^ fractions in both CD4^+^ and CD8^+^ T cells. Moreover, the model indicated that activation, proliferation and death of CD4^+^ T cells was increased, thus leading to stable or lower numbers of proliferating cells over time. On the other hand, in the CD8^+^ T-cell pool, the increased proliferation leads to larger numbers of proliferating cells [120]. These modelling results explained the telomere data: although CD4^+^ T cells have higher proliferation rates, these proliferating cells also die quickly leaving behind cells that have proliferated less, with closer to normal telomere lengths; on the other hand, proliferating CD8^+^ T cells accumulate resulting in shorter telomeres. (This possibility had already been raised by modelling of proliferation and telomere shortening [122].) In addition these models of deuterated glucose showed that, to estimate proliferation, it is important to analyse the slopes of label uptake and loss [120], rather than just the level of labelled cells at one time point [118] (figure 3).

Modelling, thus, contributed to reconcile disparate, even contradictory, results of different techniques to measure T-cell proliferation. At the same time, these initial models also suggested that analyses of this type of data may be more complex than originally thought. For example, one assumes that all cells are proliferating at the same average rate, although it is more likely that different subpopulations have different proliferation rates and/or that these change over time. This has led to numerous studies, of both modelling [79,123–128] and experimental [129–132] aspects, related to T-cell proliferation (an excellent recent review on the subject is provided in [133]).

## 6. Looking forward

Here, we have focused on examples based on T-cell immunology at the level of populations of cells to maintain a unifying theme. However, many studies from other areas of immunology would also illustrate the richness of mathematical contributions to developing insight into physiological processes. Mathematical and computational models have also been successful in humoral [134,135] and innate immunity [136,137]. The recent discovery of *de facto* innate immune memory [138,139], and of properties of tissue-resident memory T cells, reminiscent of those of innate immune cells [140], are provoking a rethink of the divisions between subfields of immunology, more in tune with the mathematician's natural urge to unite descriptions of the symphonies of molecular and cellular interactions by identifying what is essential.

These examples are often driven by technical advances in genomics, proteomics, mRNA quantification, single-cell capabilities and others, demonstrating how novel experimental approaches push model development. Indeed, new modelling approaches are also needed to make use of the complexity of the data generated [141–144]. It is important to note that these models span multiple biological scales, including intracellular and molecular levels [8,145,146]. Obviously, good mathematical models need good quantitative data, and this is often harder to obtain at finer scales, such as the molecular level. Future technological developments will bring new challenges for modellers.

In spite of these beautiful studies, collaborations between mathematical and experimental immunologists are still infrequent. These collaborations are a fertile ground for new ideas and primed for great impact; however, there are challenges that need to be taken into consideration as the field moves forward. One shared feature of emergent fields is the heterogeneity of approaches, methods and available data. In this context, a certain level of standardization could be helpful to facilitate reproducibility and model sharing and to reduce publication errors by means, for example, of language-independent model definition, with repositories of models contributed by the community. There are several such repositories, such as CellML [147], which is a free, open-source, extensible markup language-based standard for defining mathematical models of cellular function. It currently incorporates more than 50 models in the broad area of mathematical immunology. Other examples are systems biology markup language [148] and JSim [149], which allows storage of data and model analysis.

Recent technical advances permit single-cell measurements [150–152], revealing the inherent heterogeneity in immunological processes. Single-cell data interpretation is almost impossible without mathematics. To realize the full promise of mathematical immunology, more and stronger collaborations between modellers and experimental scientists are needed. One way to stimulate these is to provide venues for enhanced interactions among scientists, at international meetings such as Systems Approaches in Immunology, q-Bio conferences, the KITP Quantitative Immunology programmes, HIV Dynamics and Evolution Conference and collaborative networks such as QuanTI [153], ENLIGHT-TEN [154], INDOEUROPEAN-MATHDS [155] and INTI [156].

In conclusion, it is possible to find excellent examples of synergy between modelling and experiment in immunology, but still much remains to be done.

## Endnotes

1 This is in agreement and consistent with other single-cell tracking studies [52].

2 The superscript, (4) or (8), in a given parameter indicates that it has been estimated making use of MHC class-I- or MHC class-II-deficient mice data, respectively.
